# Quantitative Early Auxin Root Proteomics Identifies GAUT10, a Galacturonosyltransferase, as a Novel Regulator of Root Meristem Maintenance*[Fn FN2]

**DOI:** 10.1074/mcp.RA119.001378

**Published:** 2019-03-27

**Authors:** Yunting Pu, Justin W. Walley, Zhouxin Shen, Michelle G. Lang, Steven P. Briggs, Mark Estelle, Dior R. Kelley

**Affiliations:** From the Departments of ‡Genetics, Development and Cell Biology,; ¶Plant Pathology and Microbiology, Iowa State University, Ames, IA;; §Section of Cell and Developmental Biology, University of California, San Diego, La Jolla, CA

**Keywords:** *Arabidopsis*, Hormones*, Developmental Biology*, iTRAQ, Targeted Mass Spectrometry, Plant Biology*, Plant Cell Walls, Plant Stem Cells, Root Development

## Abstract

Auxin induces rapid gene expression changes throughout root development. How auxin-induced transcriptional responses relate to changes in protein abundance is not well characterized. This report identifies early auxin responsive proteins in roots at 30 min and 2 h after hormone treatment using a quantitative proteomics approach in which 3,514 proteins were reliably quantified. A comparison of the >100 differentially expressed proteins at each the time point showed limited overlap, suggesting a dynamic and transient response to exogenous auxin. Several proteins with established roles in auxin-mediated root development exhibited altered abundance, providing support for this approach. While novel targeted proteomics assays demonstrate that all six auxin receptors remain stable in response to hormone. Additionally, 15 of the top responsive proteins display root and/or auxin response phenotypes, demonstrating the validity of these differentially expressed proteins. Auxin signaling in roots dictates proteome reprogramming of proteins enriched for several gene ontology terms, including transcription, translation, protein localization, thigmatropism, and cell wall modification. In addition, we identified auxin-regulated proteins that had not previously been implicated in auxin response. For example, genetic studies of the auxin responsive protein galacturonosyltransferase 10 demonstrate that this enzyme plays a key role in root development. Altogether these data complement and extend our understanding of auxin response beyond that provided by transcriptome studies and can be used to uncover novel proteins that may mediate root developmental programs.

## Abstract (precis)

Auxin induces rapid abundance changes in various signaling proteins, transcriptional regulators, and enzymes such as cell wall modification proteins in roots. Loss of function of 15 top responsive proteins results in altered root phenotypes, demonstrating the power of this approach for reverse genetics screens. Characterization of the auxin responsive protein galacturonosyltransferase 10 demonstrates that this enzyme positively regulates sugar-mediated root meristem maintenance. Novel targeted proteomics assays demonstrate that all six auxin receptors remain stable in response to hormone.

## Graphical Abstract

**Figure:**
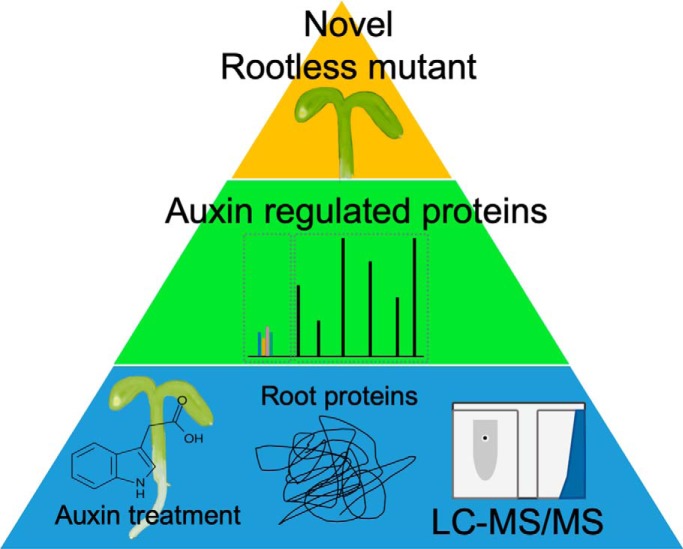

**Highlights**

- Auxin responsive proteins in *Arabidopsis* roots were identified from 3,514 detected proteins.
- All six auxin receptors are stable in response to hormone via novel MRM assays.
- The >100 differentially expressed proteins exhibit dynamic and transient responses to auxin.
- Phenotypic screening of the top responsive proteins uncovered several novel root mutants.

Auxin is a major phytohormone involved in regulating many aspects of seedling development, including cotyledon formation, hypocotyl cell elongation, meristem maintenance and root morphogenesis (1). In land plants, the response to auxin is controlled by co-receptors comprised of transport inhibitor response 1 (TIR1)/auxin F-box (TIR1/AFB)^1^ and Aux/ indole-3-acetic acid (IAA) transcriptional regulators. In *Arabidopsis*, there are 6 TIR1/AFB proteins and 29 Aux/IAA proteins (2). The Aux/IAA proteins actively repress transcription by interacting with transcription factors called auxin response factors and recruiting a co-repressor protein called TOPLESS (TPL). Auxin acts by promoting the degradation of the Aux/IAAs, leading to tightly regulated changes in gene expression that have been well documented (3–6).

One of the outstanding questions in the field is how these auxin-mediated transcriptional changes collectively influence proteome composition. *Arabidopsis* roots are an excellent model for proteomic profiling because they exhibit tissue-specific auxin responses and provide sufficient quantities of plant material for sampling. Additionally, transcriptional changes in *Arabidopsis* roots have been well documented (3, 7–9). Initial characterization of auxin-responsive proteomes in seedlings and roots identified proteins that are responsive 6–24 h after auxin treatment (10, 11) and protein phosphorylation events associated with auxin-mediated lateral root formation (12). However, further studies of the auxin-regulated proteome are needed to generate a more comprehensive view of auxin-mediated gene expression (13).

In this study, we characterized early auxin-regulated proteomes in *Arabidopsis* roots following exposure to a naturally occurring auxin, IAA, for 30 and 120 min. These data provide a proteomic description of how auxin influences early gene expression events in roots that has not been previously captured. Comparisons between differentially expressed proteins at both time points showed limited overlap, suggesting that regulation of protein abundance by auxin is dynamic. Additionally, the identification of novel auxin-regulated proteins provides the opportunity to uncover new regulators of root development. Toward this goal, we have characterized loss of function alleles of one auxin-responsive protein, galacturonosyltransferase 10 (GAUT10), for auxin-related root phenotypes. *GAUT10* mutants have short roots that are enhanced in the absence of sucrose and smaller root apical meristems. In addition, *gaut10* roots are auxin responsive in the presence of sucrose, suggesting that this protein may act downstream or independent of TIR1/AFB-Aux/IAA co-receptor action. Sugar signaling has been show to affect root growth through interactions with auxin as part of root developmental plasticity to environmental conditions. Given the potential role of *GAUT10* to directly modify pectin composition, we propose that this galacturonosyltransferase provides a novel link between cell wall modification and auxin signaling that is required for cell expansion within the developing primary root.

## EXPERIMENTAL PROCEDURES

### 

#### 

##### Plant Material

*Arabidopsis thaliana* plants used in this study were *Columbia* (Col-0) ecotype. SALK_029319 (*gaut10–1*) is a knock-out allele, and SALK_082273C (*gaut10–2*) is a knock-down allele that was previously characterized (14). SALK_092577C (*gaut10–3*) is a null allele characterized in this study. Other alleles used in this study have been previously published as null/knock-out mutants: *vps35b-1* (SALK_014345) (15); *bdx-2* (SALK_142260) (16); *camta2* (SALK_007027) (17); *gapcp2.2* (SALK_008979) (18); *atrh8* (SALK_016830) (19); *smp2* (SALK_127730) (20); *pme17–2* (SALK_059908) (21); SALK_063023 (22); *nadp-me4* (SALK_064163) (23); *rhip1–1* (SALK_091518) (24); SALK_111575 (25); *cka2–1* (SALK_129331) (26); SALK_145341 (27); SALK_151595 (28); and *impl2–3* (SAIL_35_A08) (29). For proteomics profiling, Col-0 seeds were surfaced sterilized using 50% bleach and 0.01% Triton X-100 for 10 min and then washed five times with sterile water. Seeds were then imbibed in sterile water for 2 days at 4 °C and then transferred to 0.5X Murashige-Skoog medium plates overlaid with sterile nylon mesh squares to facilitate tissue harvesting. Seedlings were grown under long day photoperiods (16 h light/8 h dark) at 23 °C. Five-day-old seedlings were treated with 1 μm IAA (“auxin”) or an equivalent volume of 95% dimethyl sulfoxide (“mock”) for 30 min or 2 h by transferring the seedlings on mesh squares to square Petri dishes containing 10 ml of fresh 0.5X MS supplemented with IAA or solvent. Following treatments, the roots were then hand dissected at the root–hypocotyl junction with a sterile scalpel, pooled to reach 1 g of tissue per biological replicate per treatment, and immediately frozen in liquid nitrogen. Four independent biological replicates were generated for each treatment and time point. For phenotyping assays seeds were surfaced sterilized using 50% bleach and 0.01% Triton X-100 for 10 min and then washed five times with sterile water. Seeds were then imbibed in sterile water for 2 days at 4 °C and then transferred to 0.5X MS medium plates. Seedlings were grown under long day photoperiods (16 h light/8 h dark) at 23 °C. For auxin response assays, 5-day-old seedlings were transferred to 0.5X MS plates supplemented with dimethyl sulfoxide or 1 μm IAA (auxin) and grown for another 2 days.

##### Root Phenotyping

Five- and 7-day-old seedlings were photographed and images were saved as JPEG files. Measurements of primary root length were calculated using the FIJI software program (https://fiji.sc/). For lateral root measurements on 7-day-old seedlings, all visible emerged lateral roots were counted. For phenotypic assays, at least 10 biological replicates were analyzed per genotype and phenotype. The root phenotyping assays (primary root length, response to auxin treatment, and lateral root formation) were repeated twice. Mutants that were significantly different in each phenotype or treatment, relative to Col-0 or mock treatment, were determined using *t*-tests (two-sample heteroscedastic); *p* values of <0.05 were considered to be significant in these assays.

##### Experimental Design and Statistical Rationale

For the iTRAQ profiling, four biological replicates were generated for each treatment and time point (*i.e.* four mock samples at 30 min, four auxin-treated samples at 30 min, four mock samples at 120 min, and four auxin-treated samples at 120 min). Each iTRAQ 4-plex set (*i.e.* 2D-LC-MS/MS run) contained one biological replicate from each treatment. Proteins that were reliably identified in three out of four biological replicates were quantified. Proteins that significantly changed in each treatment, relative to mock, were determined using *t*-tests (two-sample heteroscedastic). Proteins with a *p* value of <0.05 were considered to be significantly differentially expressed. Data analysis was performed using Perseus.

##### Preparation and Analysis of Proteins via Mass Spectrometry

Peptide preparation and protein abundance profiling by mass spectrometry are based on previously described methods (30, 31). Each frozen tissue sample was thoroughly ground to a fine powder for 15 min in liquid nitrogen prior to protein extraction. Proteins were precipitated and washed with 50 ml of −20 °C methanol three times then 50 ml of −20 °C acetone three times. Protein pellets were aliquoted into four 2-ml Eppendorf tubes and dried in a vacuum concentrator at 4 °C. Protein pellets were suspended in 1 ml of extraction buffer (0.1% SDS, 1 mm EDTA, 50 mm Hepes buffer, pH 7). Cysteines were reduced and alkylated using 1 mm of Tris (2-carboxyethyl)phosphine (Fisher, AC36383) at 95 °C for 5 min then 2.5 mm of iodoacetamide (Fisher, AC12227) at 37 °C in the dark for 15 min, respectively. Protein was quantified using a Bradford assay with bovine serum albumin used to construct the standard curve (Pierce). Proteins were digested with trypsin overnight (Roche, 03 708 969 001, enzyme:substrate w:w ratio = 1:100). A second digestion was performed the next day for 4 h (enzyme:substrate w:w ratio = 1:100). Digested peptides were purified on a 500-mg Waters Oasis MCX cartridge to remove SDS. Peptides were eluted from the MCX column with 4 ml of 50% isopropyl alcohol and 400 mm of NH_4_HCO_3_ (pH 9.5) and then dried in a vacuum concentrator at 4 °C. Peptides were resuspended in 0.1% formic acid and further purified on a 50-mg Sep-Pak C18 column (Waters). Peptide amount was quantified using the Pierce BCA Protein assay kit with bovine serum albumin used to construct the standard curve.

**Fig. 1.**
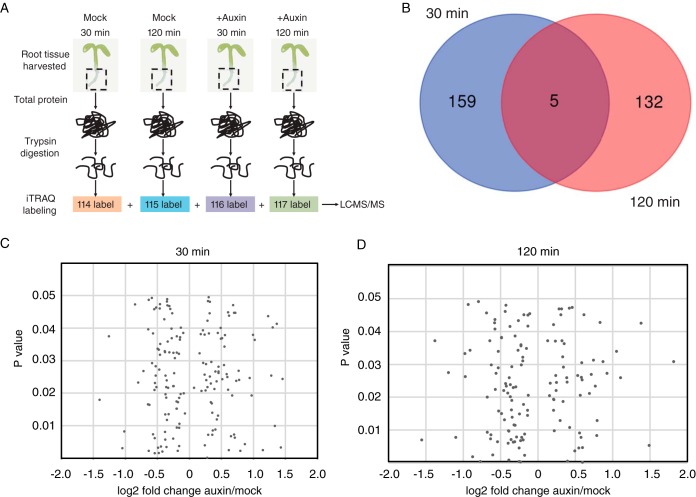
**Quantitative proteomic analysis of early auxin-responsive proteomes in *Arabidopsis* roots identifies ∼300 differentially expressed proteins.** (*A*) Schematic of the experimental workflow. Five-day-old wild-type seedlings were treated with 1 μm IAA (auxin) or an equivalent volume of solvent control (mock) for 30 min and 120 min, and dissected roots were processed for proteome profiling using four-plex iTRAQ labeling as diagramed. This was repeated three more times for a total of four multiplexed 2D-LC-MS/MS runs (*B*) Only five proteins are differentially expressed at both time points (*p* value ≤ 0.05). 164 differentially expressed proteins (*p* value ≤ 0.05) detected at 30 min (*C*) and 137 proteins at 120 min (*D*).

Peptides were labeled with iTRAQ reagents (AB SCIEX) (Fig. 1) according to the following scheme per biological replicate: 114—mock 30-min sample, 115—mock 120-min sample, 116—auxin 30-min sample, and 117—auxin 120-min sample. This was repeated three more times for a total of four multiplexed runs. We obtained higher than 95% iTRAQ labeling efficiency by treating 100 μg of nonmodified peptides with one tube of iTRAQ reagent for 2 h at room temperature. Labeled samples were dried down in a vacuum concentrator and resuspended in 0.1% formic acid. Samples tagged with the four different iTRAQ reagents were pooled together.

An Agilent 1200 HPLC system (Agilent Technologies) was used to deliver a flow rate of 600 nl/min via a three-phase capillary chromatography column through a splitter to the mass spectrometer. The three-phase capillary chromatography was set up as follows. Using a custom pressure cell, 5-μm Zorbax SB-C18 (Agilent) was packed into fused silica capillary tubing (200-μm inner diameter, 360-μm outer diameter, 30-cm long) to form the first dimension reverse-phase column (RP1). A 5-cm long strong cation exchange column packed with 5-μm PolySulfoethyl (PolyLC) was connected to RP1 using a zero dead volume 1-μm filter (Upchurch, M548) attached to the exit of the RP1 column. A fused silica capillary (200-μm inner diameter, 360-μm outer diameter, 20-cm long) packed with 2.5-μm C18 (Waters) was connected to strong cation exchange as the analytical column (RP2). The electrospray tip of the fused silica tubing was pulled to a sharp tip with the inner diameter smaller than 1 μm using a laser puller (Sutter P-2000). The peptide mixtures were loaded onto the RP1 column using the custom pressure cell. A new set of columns was used for each LC-MS/MS analysis. Peptides were first eluted from the RP1 column to the strong cation exchange column using a 0% to 80% acetonitrile gradient for 60 min. The peptides were then fractionated by the strong cation exchange column using a series of 27 salt steps for nonmodified iTRAQ profiling (20, 40, 50 55, 60, 62.5, 65, 67.5, 70, 72.5, 75, 77.5, 80, 82.5, 85, 87.5, 90, 92.5, 95, 97.5, 100, 120, 150, 180, 200, 500, 1,000 mm ammonium acetate) followed by high-resolution reverse-phase separation using an acetonitrile gradient of 0 to 80% for 150 min.

Spectra were acquired using an LTQ Velos linear ion trap tandem mass spectrometer (Thermo Electron Corporation, San Jose, California) employing automated, data-dependent acquisition. The mass spectrometer was operated in positive ion mode with a source temperature of 250 °C. The full MS scan range of 400–2,000 *m/z* was divided into three smaller scan ranges (400–800, 800–1,050, 1,050–2,000) to improve the dynamic range (32–34). Both collision-induced dissociation and pulsed-Q dissociation scans of the same parent ion were collected for protein identification and quantitation. Each MS scan was followed by four pairs of collision-induced dissociation–pulsed-Q dissociation MS/MS scans of the most intense ions from the parent MS scan. A dynamic exclusion of 1 min was used to improve the duty cycle of MS/MS scans.

The raw data were extracted and searched using Spectrum Mill v3.03 (Agilent). The collision-induced dissociation and pulsed-Q dissociation scans from the same parent ion were merged together. MS/MS spectra with a sequence tag length of 1 or less were considered to be poor spectra and were discarded. The remaining MS/MS spectra were searched against The Arabidopsis Information Resource (TAIR), *Arabidopsis* TAIR10 database, which contains 70,800 protein sequences in the database. The enzyme parameter was limited to fully tryptic peptides with a maximum miscleavage of 1. All other search parameters were set to default settings of Spectrum Mill (carbamidomethylation of cysteines and iTRAQ modification). Carbamidomethylation of cysteines and iTRAQ modifications as the fixed modifications and Ox-Met and n-term pyro-Gln as the variable modifications. Mass tolerances were ± 2.5 Da for precursor ions and ± 0.7 Da for fragment ions. A concatenated forward-reverse database was constructed to calculate the *in situ* false discovery rate. All datasets were summarized together to maintain false discovery rates across the datasets. Cutoff scores were dynamically assigned resulting in false discovery rates of 0.05%, 0.12%, and 0.66% at the spectrum, peptide, and protein level, respectively. Proteins that share common peptides were grouped to address the database redundancy issue. The proteins within the same group shared the same set or subset of unique peptides.

iTRAQ intensities were calculated by summing the peptide iTRAQ intensities from each protein group. Peptides shared among different protein groups were removed before quantitation using custom Perl scripts implemented in Spectrum Mill v3.03 (Agilent). Isotope impurities of iTRAQ reagents were corrected using correction factors provided by the manufacturer (Applied Biosystems). Median normalization was performed to normalize the protein iTRAQ reporter intensities in which the log ratios between different iTRAQ tags (115/114, 116/114, 117/114) are adjusted globally such that the median log ratio is zero. Protein ratios between the mock and each treatment were calculated by taking the ratios of the total iTRAQ intensities from the corresponding iTRAQ reporter. Protein ratios were then log_2_ converted. Data analysis was performed using Perseus.

##### Development and Analytical Validation Targeted MS Assays/Measurements

“Tier 2” targeted MS assays were developed to detect and quantify seven endogenous proteins (six auxin receptor proteins and one actin control protein) from *Arabidopsis* root tissues. These multiple reactions monitoring (MRM) assays were designed and performed according to (32) with the following modifications. Reference peptides were designed for actin and TIR1/AFB proteins using Skyline (Table S5). The actin peptide (VAPEEHPVLLTEAPLNPK) was used for normalization of sample loading and therefore designed to detect multiple actin proteins (AT2G42170, AT5G59370, AT3G46520, AT3G12110, AT5G09810). A synthetic plasmid (pDK185) containing these peptide sequences separated by lysine residues (supplemental Fig. S3) was generated from Genewiz by insertion of the chimeric DNA sequence into a pUC57 backbone. This chimeric sequence was then cloned into pT7CFE1-CGST-HA-His (Thermo Scientific) using BamHI and SalI sites, generating plasmid pDK186. Heavy-labeled reference peptides were then generated by *in vitro* transcription translation using the 1-Step Heavy Protein IVT kit (Thermo Scientific). Peptides were purified using His-tag Dynabeads (Invitrogen) and subjected to on-bead digestion with trypsin to recover heavy-labeled reference peptides; 2 μl of in vitro transcription (IVT) peptides were spiked into samples. Method optimization was performed to determine the optimal transitions and collision energies for each protein. Three to four biological replicates were assayed for each treatment/time point and two to three technical replicates were run for each biological replicate (21 samples in total). Digested peptides were injected by Agilent 1200 autosampler and subjected to a 40-min reverse-phase separation. The intensities from the best transitions were used to quantify relative abundance of TIR1, AFB1, AFB2, AFB3, AFB4, and AFB5 in auxin-treated samples compared with mock-treated samples (Table S5, Fig. S3). Interference-free transitions with high intensities were selected for quantification in Skyline (Fig. S3).

##### Gene Ontology (GO) Enrichment

GO enrichment analyses were performed using PANTHER 13.1 using *A. thaliana* as the organism (http://pantherdb.org/). A heatmap of enriched GO biological processes was generated following hierarchical clustering in MeV.

##### Confocal Imaging and Propidium Iodide Staining

Confocal imaging of roots was performed using a Leica SP5 X MP confocal/multiphoton microscope system at the Iowa State University Microscopy and Nanoimaging Facility. Prior to confocal imaging, seedlings were stained with 10 mg/ml propidium iodide for 1–3 min, rinsed in water, and then imaged under 40x/1.25 oil immersion objective with excitation wavelength of 488 nm and emission wavelength of 500–550 nm.

## RESULTS

### 

#### 

##### Quantitative Proteomic Analysis of Roots Following Auxin Treatments Identifies Dynamic Changes

The transcriptional responses to exogenous auxin in *Arabidopsis* roots have been well characterized (3, 8, 36). We sought to extend these studies by examining early proteome changes in *Arabidopsis* roots associated with auxin using mass spectrometry. Thus, we selected 30 min and 2 h as “early” time points to profile auxin-regulated proteins (Fig. 1*A*). A total of 5,312 proteins were detected, and 3,514 proteins were reliably quantified (*i.e.* detected in at least three out of four biological replicates) (Table S1).

**Fig. 2.**
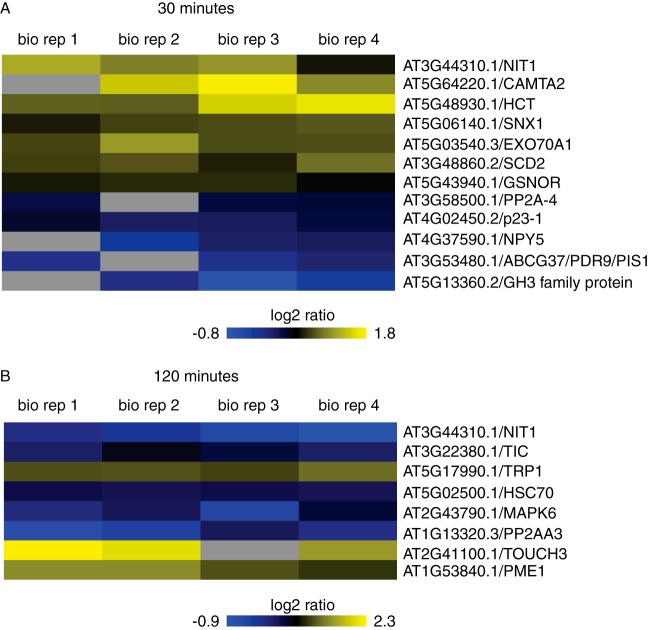
**Several well-characterized proteins involved in various auxin pathways exhibit modest but significant DE in roots (*p* value ≤ 0.05) following 30 min (*A*) and 120 min (*B*) of exogenous auxin treatment.** This includes nitrilase 1, calmodulin-binding transcription activator 2 (CAMTA2), hydroxycinnamoyl-CoA shikimate/quinate hydroxycinnamoyl transferase (HCT), SNX1, exocyst subunit exo70 family protein A1 (EXO70A1), stomatal cytokinesis defective 2 (SCD2), GroES-like zinc-binding dehydrogenase family protein (GSNOR), protein phosphatase 2A-4 (PP2A-4), p23–1 (HSP20-like chaperones superfamily protein), naked pins in YUC mutants 5 (NPY5), ATP-binding cassette G37/pleiotropic drug resistance 9/polar auxin transport insensitivePOLAR 1 (ABCG37/PDR9/PIS1), an auxin-responsive GH3 family protein, time for coffee (TIC), tryptophan biosynthesis 1 (TRP1), HSP70, MAP kinase 6 (MAPK6), protein phosphatase 2A subunit A3 (PP2AA3), TOUCH3, and pectin methylesterase 1 (PME1). Heatmap indicates the log2 fold change of auxin/mock for all four biological replicates; increased abundance is indicated in yellow while reduced protein abundance is indicated in blue.

At 30 min, 164 proteins are differentially expressed relative to the mock control while 137 proteins are differentially expressed at 2 h (*p* value ≤ 0.05) (Fig. 1*B*). In general, most of the significantly differentially expressed proteins exhibited modest fold change (FC) values (Figs. 1*C* and 1*D*, Table S1), which is in line with the observed ratio compression associated with isobaric tags for relative and quantitative abundance (iTRAQ) methodology (37–41). Specifically, the observed fold changes in iTRAQ data are often compressed and may lead to underestimation of relative protein levels. Notably, a number of the top responsive proteins (log_2_ FC ≥0.58 or ≤0.58) have known roles in auxin-mediated pathways, thus their modest change in auxin-driven protein abundance is likely sufficient for driving phenotypic changes (Fig. 2). The “top 10” responsive proteins in each category (up or down) and timepoint (30 and 120 min) are shown in Table 1.

**Table I:** “Top 10” auxin-responsive proteins in Arabidopsis roots. DE proteins (p value < 0.05) were filtered for a log2 FC cutoff of 0.58 or greater (i.e. a 1.5 FC or greater) either increased or decreased in abundance in the auxin-treated samples compared to mock. Proteins are ranked according to descending FC values

Category	Locus	Protein name/description	Log2 FC
30 min up	AT1G69740.1	Aldolase superfamily protein (HEMB1)	1.69
30 min up	AT5G21105.1	Plant l-ascorbate oxidase	1.46
30 min up	AT5G09620.1	Octicosapeptide/Phox/Bem1p family protein	1.43
30 min up	AT5G15450.1	Albino and pale green 6 (APG6); casein lytic proteinase B3 (CLPB3)	1.37
30 min up	AT5G35180.1	Enhanced disease resistance proteing (DUF1336)	1.34
30 min up	AT5G64220.1	Calmodulin-binding transcription activator 2 (CAMTA2)	1.30
30 min up	AT3G48890.1	Membrane-associated progesterone binding protein 3 (MAPR3)	1.28
30 min up	AT5G66720.2	Protein phosphatase 2C family protein	1.23
30 min up	AT1G23870.1	Trehalose-phosphatase/synthase 9 (ATTPS9/TPS9)	1.15
30 min up	AT5G48930.1	Hydroxycinnamoyl-CoA shikimate/quinate hydroxycinnamoyl transferase (HCT)	1.02
30 min down	AT3G20250.1	Pumilio 5 (PUM5)	−1.40
30 min down	AT4G00660.2	RNAhelicase-like 8 (RH8/ATRH8)	−1.25
30 min down	AT4G39120.1	Myo-inositol monophosphatase like 2 (IMPL2/HISN7)	−1.04
30 min down	AT1G20696.3	High mobility group B3 (HMGB3/NFD3/NFD03)	−1.01
30 min down	AT3G17840.1	Receptor-like kinase 902 (RLK902)	−0.85
30 min down	AT3G02710.1	ARM repeat superfamily protein	−0.80
30 min down	AT4G00752.1	UBX domain-containing protein	−0.71
30 min down	AT5G14540.1	Basic salivary proline-rich-like (DUF1421)	−0.64
30 min down	AT5G17410.2	Spc97/Spc98 family of spindle pole body (SBP) component	−0.64
30 min down	AT5G14030.2	Translocon-associated protein beta (TRAPB) family protein	−0.63
120 min up	AT2G41100.1	TOUCH3/calmodulin-like 12 (TCH3/CML12)	1.83
120 min up	AT5G06200.1	Casparian strip membrane domain protein 4 (CASP4)	1.50
120 min up	AT1G53710.1	Calcineurin-like metallo-phosphoesterase superfamily protein	1.39
120 min up	AT5G05780.1	Asymmetric leaves enhancer 3/RP non-ATPase subunit 8A (AE3/RPN8A)	1.11
120 min up	AT4G19610.1	Nucleotide/RNA binding protein	1.06
120 min up	AT3G54860.2	Vacuolar sorting protein 33 (VPS33)	0.93
120 min up	AT5G09390.1	CD2-binding protein-related	0.88
120 min up	AT3G50000.1	Casein kinase II, alpha chain 2 (CKA2)	0.84
120 min up	AT5G01390.4	DNAJ heat shock family protein	0.80
120 min up	AT1G79750.1	NADP-malic enzyme 4 (NADP-ME4)	0.79
120 min down	AT2G43680.1	IQ-domain 14 (IQD14)	−1.55
120 min down	AT3G02480.1	ABA-RESPONSIVE PROTEIN (ABR)	−1.37
120 min down	AT4G31790.2	Tetrapyrrole (Corrin/Porphyrin) Methylases	−1.19
120 min down	AT4G32460.2	BIIDXI (BDX)	−1.09
120 min down	AT2G20810.1	Galacturonosyltransferase 10 (GAUT10/LGT4)	−0.97
120 min down	AT1G16300.1	Glyceraldehyde-3-phosphate dehydrogenase of plastid 2 (GAPCP-2)	−0.97
120 min down	AT4G27960.2	Ubiquitin conjugating enzyme 9 (UBC9)	−0.93
120 min down	AT3G05680.1	Virilizer (VIR), embryo defective 2016 (EMB2016)	−0.90
120 min down	AT5G40390.1	Raffinose synthase 5 (RS5), seed imbibition 1-like (SIP1)	−0.87
120 min down	AT4G37120.1	SWELLMAP 2 (SMP2)	−0.79

While 3,419 proteins were detected in at least three out of the four replicates at both time points (Table S1), only five proteins are differentially expressed at both time points (Fig. 1*D*). This may indicate that auxin-mediated changes in root proteomes are rapid and transient. Proteins common to both time points include nitrilase 1 (At3g44310), mucilage-modified 2 (At5g63800), a putative eukaryotic elongation factor 1A (eEF1A), a methyltransferase (At1g66680), eukaryotic translation initiation factor isoform 4G1 (At5g57870), and an unknown protein (At3g03150). Nitrilase 1 regulates root growth and development through modulation of auxin metabolism (42) and was previously shown to be differentially expressed in roots at later time points following auxin treatment using iTRAQ (11). These other common auxin-responsive proteins do not yet have established roles in auxin signaling.

##### Key Auxin-responsive Proteins are Dynamically Regulated

We examined the differentially expressed proteins in more detail in order to identify particular proteins that may play known roles in auxin biology. Proteins with altered abundance levels in auxin-treated roots have been previously linked to auxin pathways, providing support for these profiling data (Fig. 2 and Table S1). For example, this group includes several proteins associated with auxin transport in roots, such as sorting nexin 1 (SNX1), time for coffee (TIC), MAP kinase 6 (MAPK6), protein phosphatase 2A A3 (PP2AA3), TOUCH3, and exocyst subunit exo70 family protein A1 (EXO70A1) (Fig. 2). SNX1 has been previously reported to increase in both abundance and phosphorylation in roots following auxin treatment (12), which is consistent with the modest increase in abundance observed in our root dataset. SNX1 is a key component of the retromer complex that acts to retrieve the pin-formed (PIN) family of auxin transporters from late/prevacuolar compartment back to recycling pathways and fine tunes auxin responses during gravitropism (12, 43–46). TIC has a role in controlling root meristem size, reduced PIN expression, and acropetal auxin transport in *tic-2* mutants (47). MAPK6 has been shown to regulate postembryonic root development and auxin levels (48), while MAPK6 activity is correlated with repression of primary root growth, and auxin signaling induces MAPK6 activity (49). In our datasets, we only observed unmodified (*i.e.* unphosphorylated) levels of MAPK6 and observed down-regulation at 120 min (Fig. 2*B*). PP2AA3 regulates auxin distribution and stem cell function at the root apex through interaction with PIN proteins (50, 51). TOUCH3 (52) interacts physically with PINOID (52), and TOUCH3 expression was speculated to be under the influence of auxin (53). In these data, TOUCH3 levels increase 120 min following auxin treatment in roots (Fig. 2), which is consistent with these published reports. Finally, EXO70A1 is of interest because the exocyst is involved in PIN1 and PIN2 recycling and thus contributes to polar auxin transport regulation (54). Modest up-regulation of EXO70A1 occurs at 30 min after auxin treatment (Fig. 2*A*), which is in line with timing observed for PIN1 and PIN2 recycling. Finally, ATP-binding cassette G37/pleiotropic drug resistance 9/polar auxin transport insensitive 1 (ABCG37/PDR9/PIS1) regulates auxin distribution and homeostasis in roots by excluding indole butyric acid from the root apex (55–57) and was observed to be downregulated in roots 30 min after exogenous auxin exposure, suggesting rapid feedback on auxin homeostasis pathways (Fig. 2).

##### Auxin Receptors are Stable in Response to Hormone

Auxin perception by the TIR1/AFB-Aux/IAA families of co-receptor complexes is central to auxin response factors action. However, none of these proteins were among the differentially expressed protein lists. This could be due to lack of detection via MS or other reasons. Further examination of the detected proteins revealed that only AFB1 (At4g03190) was detected in these datasets and was not found to be auxin responsive. In order to verify these results, we developed a multiplexed targeted proteomics assay to simultaneously quantify all six auxin receptor proteins and an actin control protein using heavy-labeled synthetic proteotypic peptides (supplemental Figs. S2 and S3). From these assays, we were able to confirm our iTRAQ results for AFB1 (Fig. S2). Additionally, all of the six auxin receptors appear to be stable in the presence of 1 μm IAA at 30 and 120 min.

##### Mutant Validation of Top Responsive Proteins

In order to further verify the biological relevance of the identified differentially expressed proteins, we performed phenotypic assays. We examined 17 of the top responsive proteins that have not been previously phenotyped for auxin-mediated root growth for analysis (Table II). All alleles used for these assays have been previously published as null/strong alleles (see Experimental Procedures for references). We examined three phenotypes: (1) primary root length in 5-day-old seedlings, (2) primary root inhibition following auxin treatment, and (3) lateral root formation in 7-day-old seedlings, both in the absence and presence of auxin. In 5-day-old seedlings, 9/17 lines exhibited shorter root lengths that were statistically different compared with wild-type Col-0 (Table II, “Root length” column), which equates to 53% of the proteins tested as displaying primary root phenotypes. In young *Arabidopsis* seedlings, auxin treatment can inhibit primary root growth. To test the auxin responsiveness of these mutant lines, we grew them on 0.5X MS for 5 days and then transferred them to either 0.5X MS control plates or plates supplemented with 1 μm IAA for two more days; root length was measured before and after the treatments. Relative root growth on auxin was calculated as the percentage relative to untreated seedlings. Col-0 has a 78% reduction in root growth in response to auxin (Table II, “% Root growth” column). Notably, one mutant, *gapcp2.2* (SALK_008979), exhibited auxin insensitivity while all the other mutants tested had a normal response to auxin with respect to primary root inhibition. Finally, auxin is a positive regulator of lateral root formation. Subsequently, we also examined lateral root formation phenotypes in response to auxin treatment in these same seedlings. In order to account for differences in root length, we calculated the number of lateral roots per millimeter of root length. Col-0 produces 0.23 ± 0.01 lateral roots/mm of root length following 2 days of auxin treatment. In comparison, 13/17 mutants exhibited reduced lateral root formation; 2/17 mutants failed to form any lateral roots and were thus auxin insensitive in this assay. Altogether, 76% of the top responsive proteins tested had auxin-mediated lateral root defects. Overall, identification of >50% root and/or auxin-regulated phenotypes via reverse genetics is a significant validation of these data.

**Table II:** Root phenotyping for 16 top responsive candidate proteins that exhibit differential expression following auxin treatment. Primary root lengths were measured at 5 and 7 days after germination. For auxin response assays, seedlings (n > 10) were grown for 5 days and then transferred to either control plates (0.5X MS) or 0.5X MS plates supplemented with 1 μm IAA and allowed to grow for two more days. Primary root length and lateral root number were subsequently calculated on treated seedlings. S.E. = standard error. p values were calculated using two-tailed t tests of unequal variance. Significant phenotypes are indicated in bold

Genotype	Allele	Protein ID	Root length (mm) +/− S.E.	*p* value	Primary root phenotype	% Root growth on auxin relative to untreated	*p* value	No. lateral roots/mm on auxin +/− S.E.	*p* value	Lateral root phenotype on auxin
Col-0			19.878 +/− 0.483	n/a	Wild-type	78%		0.23 +/− 0.01	Wild-type	
SALK_029319	*gaut10–1*	AT2G20810.1	17.821 +/− 0.903	0.054	**Short roots**	103%	0.39	**0.08 +/− 0.02**	**0.000029**	**Reduced lateral roots**
SALK_014345	*vps35b-1*	AT1G75850.1	**15.336 +/1 1.387**	**0.013**	**Short roots**	126%	0.45	0.22 +/− 0.03	0.768	Wild-type
SALK_142260	*bdx-2*	AT4G32460.2	19.619 +/− 0.874	0.796	Wild-type	74%	0.67	**0.11 +/− 0.02**	**0.0006**	**Reduced lateral roots**
SALK_007027	*camta2*	AT5G64220.1	**15.286 +/− 0.658**	**0.000002**	**Short roots**	73%	0.63	**0.10 +/− 0.02**	**0.00007**	**Reduced lateral roots**
SALK_008979	*gapcp2.2*	AT1G16300.1	20.024 +/− 0.928	0.890	Wild-type	**138%**	**0.001**	**0.01 +/− 0.008**	**8.20E-07**	**No lateral roots**
SALK_016830	*atrh8*	AT4G00660.2	**13.714 +/− 1.592**	**0.002**	**Short roots**	77%	0.95	**0.07 +/− 0.03**	**0.0002**	**Reduced lateral roots**
SALK_127730	*smp2*	AT4G37120.1	**15.904 +/− 0.755**	**0.0001**	**Short roots**	84%	0.53	**0.07 +/− 0.02**	**6.54E-06**	**Reduced lateral roots**
SALK_059908	*pme17–2*	AT2G45220.1	**17.600 +/− 0.716**	**0.012**	**Short roots**	79%	0.84	**0.07 +/− 0.03**	**0.0001**	**Reduced lateral roots**
SALK_063023		AT5G09620.1	19.602 +/− 0.747	0.758	Wild-type	70%	0.51	**0.05 +/− 0.02**	**5.40E-06**	**Reduced lateral roots**
SALK_064163	*nadp-me4*	AT1G79750.1	18.954 +/− 1.325	0.520	Wild-type	76%	0.91	0.16 +/− 0.03	0.07	Wild-type
SALK_091518	*rhip1–1*	AT4G26410.1	20.885 +/− 1.14	0.425	Wild-type	86%	0.45	**0.17 +/− 0.03**	**0.02**	**Reduced lateral roots**
SALK_111575	*clpb3*	AT5G15450.1	**17.512 +/− 0.814**	**0.017**	**Short roots**	87%	0.49	**0.13 +/− 0.04**	**0.03**	**Reduced lateral roots**
SALK_129331	*cka2–1*	AT3G50000.1	18.683 +/− 0.885	0.246	Wild-type	68%	0.19	**0.13 +/− 0.02**	**0.001**	**Reduced lateral roots**
SALK_145341	*abr*	AT3G02480.1	**17.181 +/− 0.902**	**0.018**	**Short roots**	75%	0.80	0.22 +/− 0.03	0.181	Wild-type
SALK_151595	*ae3/rpn8a*	AT5G05780.1	**15.824 +/− 0.910**	**0.001**	**Short roots**	71%	0.49	**0 +/− 0**	**4.90E-08**	**No lateral roots**
SAIL_35_A08	*impl2–3*	AT4G39120.1	18.907 +/− 1.206	0.461	Wild-type	85%	0.72	**0.11 +/− 0.02**	**0.0001**	**Reduced lateral roots**

##### Auxin-responsive Proteins Fall into Diverse Functional Categories

**Fig. 3.**
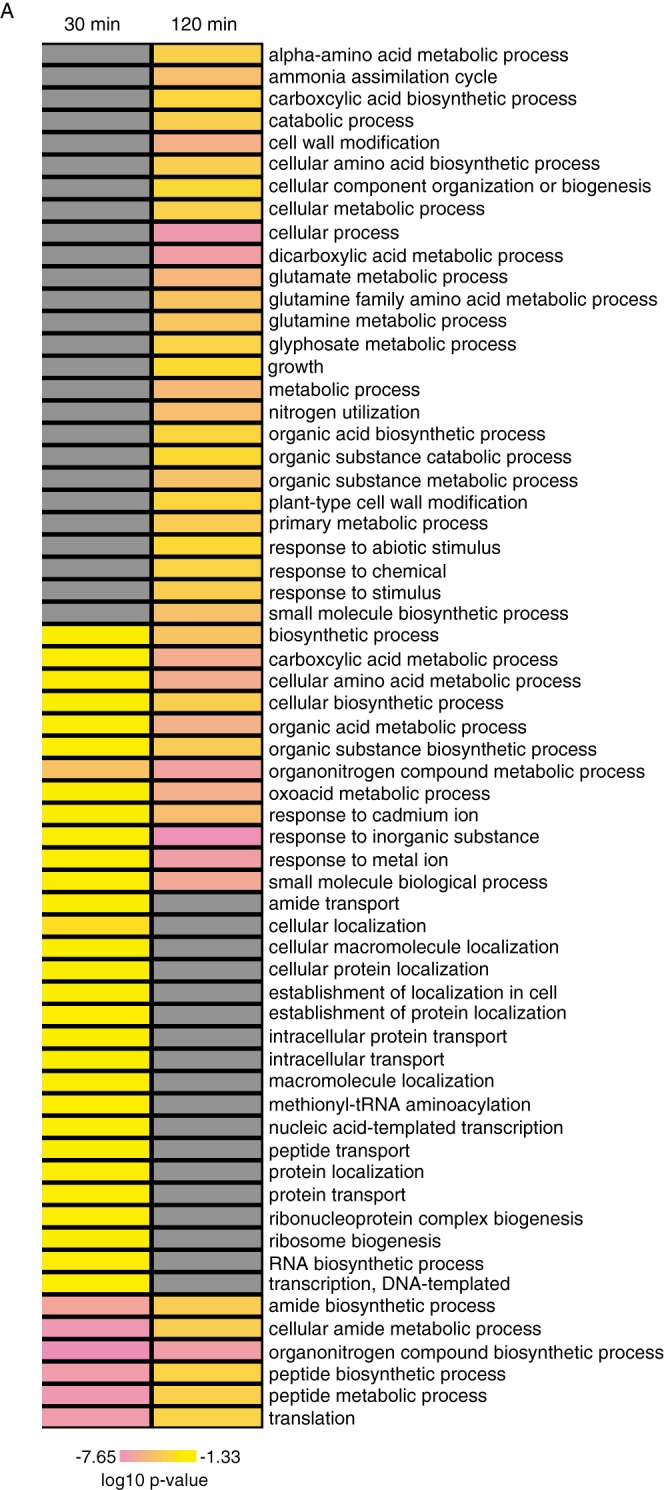
**Auxin-regulated proteins are enriched in several GO biological process categories; log10 *p* values as indicated in the color scale (gray boxes indicate not significantly enriched) (*A*) Hierarchical clustering of GO functional categories enriched in differentially expressed (DE) proteins following 30 min and 120 min of auxin treatment in roots.** Categories in common to both time points include amino acid metabolism, response to metal ions, and translation. Categories unique to the 30-min DE proteins include protein localization and transcription while cell wall metabolism and growth are enriched in the 120 min dataset.

Most of the auxin-regulated proteins were distinct between the two time points (Fig. 1), leading us to hypothesize that they may have different biological functions. In order to test this idea we performed GO enrichment analysis on the differentially expressed proteins (Fig. 3, Fig. S3, Table S2). Several enriched GO categories are common to both time points, which suggests that, while the individual proteins may vary, the overall biological processes that are impacted by auxin signaling are retained (Fig. 3, Fig. S3, Table S2). Such categories include “translation (GO:0006412),” “cellular amino acid metabolic process (GO:0006520),” “response to cadmium ion (GO:0046686),” and “response to heavy metal ion (GO:0010038)” (Fig. 3). The interaction between auxin homeostasis and heavy metal ion toxicity (including cadmium) is of interest given the widespread nature of this environmental stress. This result is in line with published studies indicating auxin metabolism and polar transport pathways can be modulated by heavy metal stimuli (58–61).

Additionally, a couple notable GO biological processes are enriched temporally in these data. For example, after 30 min of auxin treatment, GO categories related to transcription, protein localization, and microtubule dynamics are enriched (Fig. 3, Fig. S3) which is consistent with current models for early downstream auxin signaling events involving active regulation of transcription and organization of cellular transporters and actin (reviewed in (62)). Whereas cell wall modification (GO:0042545), growth (GO:0040007), and thigmotropism are enriched after 120 min of auxin treatment (Fig. 3, Fig. S3), which would fit well with the timing related to these processes.

##### A Galacturonosyltransferase Protein, GAUT10, is Auxin Regulated and Required for Root Development

We wanted to explore these datasets to uncover novel proteins downstream of auxin co-receptor action that may mediate root developmental programs. One of the top auxin-responsive proteins is GAUT10 (Table I; ranked #5 in down-regulated proteins after 120 min). GAUT10 has been implicated in pectin biosynthesis as *gaut10* alleles have reduced altered glycosyl residue compositions compared with wild type, including reduced levels of galacturonic acid in seedlings (14). Auxin-mediated cell expansion has long been linked to cell wall mechanics, and thus we hypothesized that down-regulation of GAUT10 levels may be involved in such a process. Additionally, “cell wall modification” was one of the GO-enriched biological process terms (Fig. 3, Fig. S3) adding further support for testing this candidate protein.

**Fig. 4.**
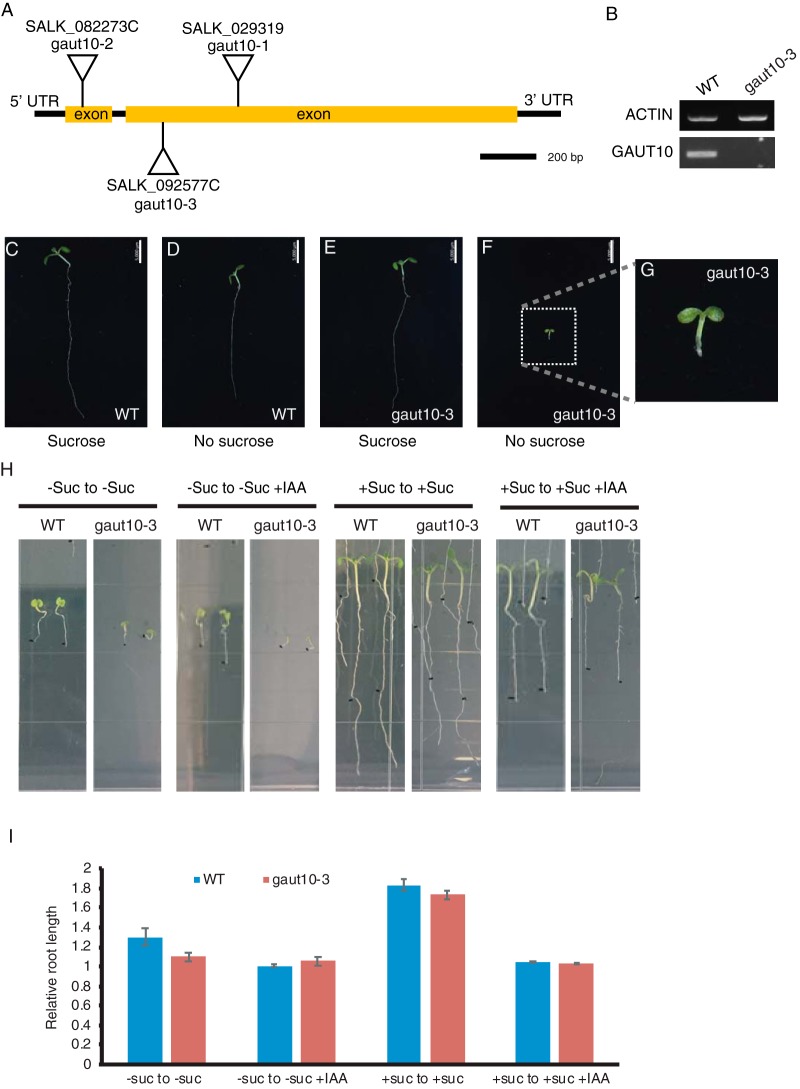
**Loss of function *gaut10* allele *gaut10–3* have short roots in the absence of sucrose but retain auxin responsiveness.** (*A*) SALK T-DNA insertion alleles of *GAUT10. gaut10–1* (SALK_029319) and *gaut10–2* (SALK_082273C) were previously characterized as knock-out and knock-down alleles, respectively (14). (*B*) RT-PCR analysis of *gaut10–3* (SALK_092577C) indicates it is a null allele of GAUT10. (*C–G*) Five-day-old seedlings of wild-type and *gaut10–3* grown with or without sucrose. (*H*) *gaut10–3* roots are shorter than wild type in the absence of sucrose but can still respond normally to exogenous auxin (IAA) treatment when grown on sucrose as indicated by lack of primary growth past the black mark and induced lateral root formation. (*I*) Quantification of inhibition of primary root growth by exogenous auxin. Scale bars shown in *C–F* is 5 mm.

In order to test this idea, we performed functional characterization of *gaut10* mutants with respect to root development and auxin response. In addition to the previously published *gaut10* alleles, *gaut10–1* and *gaut10–2* (14), we characterized an additional T-DNA insertional allele, SALK_092577C, as a null and designated it *gaut10–3* (Figs. 4*A* and 4*B*). During the process of growing these mutant alleles we observed a sucrose-dependent short root phenotype (Figs. 4*C*-4*G*). While light-grown wild-type *Arabidopsis* roots have somewhat impaired growth on MS media lacking sucrose due to arrest of the root apical meristem (RAM) (63, 64), *gaut10* mutants are hypersensitive to sucrose-deficient media compared with wild type.

To test whether *gaut10* mutants remain normal auxin response, we performed auxin response assays on wild-type and *gaut10* seedlings. For these experiments, seedlings were grown on 0.5X MS without sucrose or with 1% sucrose media for 5 days and imaged (Fig. 4*H*, *left panels*). Seedlings were then transferred to fresh 0.5X MS without sucrose or with 1% sucrose media plates supplemented with dimethyl sulfoxide or 1 μm IAA (Fig. 4*H*, *right panels*). After two additional days of growth, seedlings were reimaged, and primary root length was measured again. The ratio of primary root length posttreatment to pretreatment of auxin was calculated from three independent replicate experiments with at least nine seedlings measured per genotype each condition (Fig. 4*I*). When grown on sucrose or without sucrose, the ratio of root elongation in *gaut10–3* are similar to wild type, indicating inhibition of primary root growth following 2 days of exogenous auxin treatment (Fig. 4*I*), suggesting *gaut10* roots exhibit normal response to auxin. Additionally, exogenous auxin treatment cannot overcome the short root phenotype of *gaut10* seedlings when grown without sucrose.

**Fig. 5.**
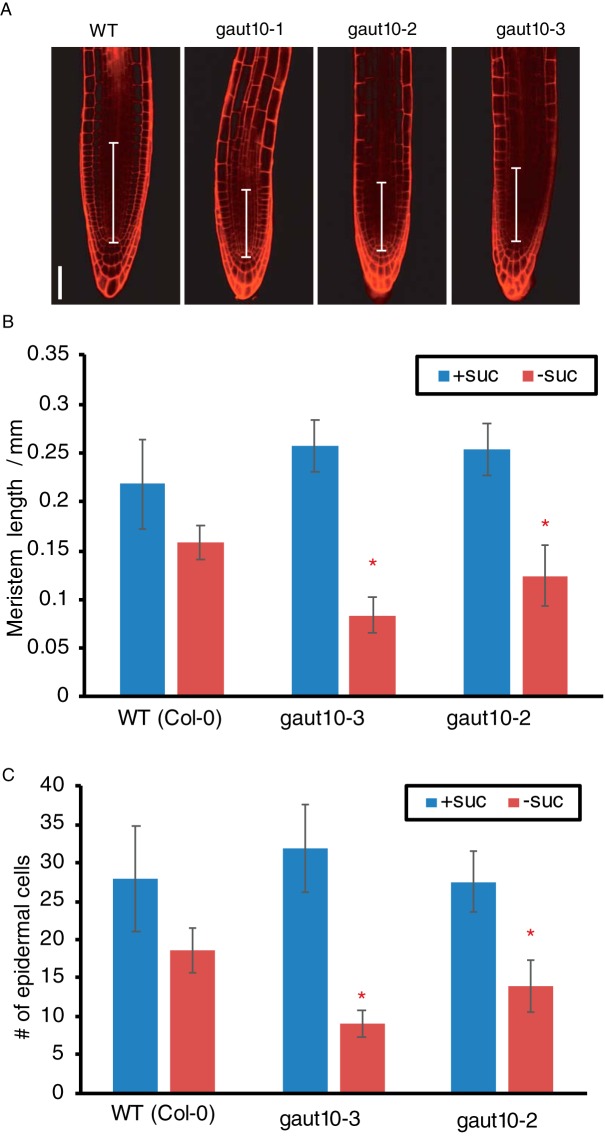
***gaut10* mutant roots have a shorter root apical meristem compared with wildtype.** (*A*) Confocal images of propidium iodide stained 5-day-old roots of wild type and *gaut10* alleles grown on media without sucrose. The *gaut10* alleles have smaller root apical meristem size based on the distance between the quiescent center (QC) and the beginning of the elongation zone (indicated by a white bar in each root). Scale bar = 50 μm. All images were acquired at the same magnification. (*B* and *C*) Quantification of (*A*), the smaller root apical meristems of *gaut10 alleles* are due to (*B*) a shorter meristem length and (*C*) fewer cells in the meristem and transition zone. Asterisk indicates statistical significance as determined by the *p* value for each comparison as determined by *t* tests.

Short root phenotypes can manifest due to RAM arrest, lack of cell elongation and/or a reduction in cell number. Through confocal imaging of 5-day-old seedlings, we observed that both *gaut10* alleles, *gaut10–2* and *gaut10–3*, appeared to have a shorter RAM in roots compared with wild-type roots when grown in the absence of sucrose (Fig. 5*A*). The distance between the quiescent center and elongation zone appeared to be shorter in *gaut10* roots than wild-type (Figs. 5*A* and 5*B*). We also counted the number of visible epidermal cells along one side of each root from the quiescent center to the first elongated epidermal cell (Fig. 5*C*), which indicated that *gaut10* RAM contains fewer cells in the absence of sucrose. Thus, loss of *gaut10* leads to shorter RAM, which underlies the short root phenotype in the absence of sucrose.

## DISCUSSION

The effects of auxin on gene regulation have been well appreciated at the transcriptional level. In this study, we describe rapid and quantitative auxin-dependent proteome changes that occur in *Arabidopsis* roots using quantitative proteomics. These datasets show that auxin-regulated proteins belong to diverse functional categories such as amino acid metabolism, RNA and protein regulation, and cell wall modification, which is consistent with the conventional wisdom that auxin-signaling impacts many aspects of plant growth and development. Additionally, auxin-responsive proteins exhibit a degree of temporal specificity as very few auxin-responsive proteins in roots are found in common between 30 min and 120 min following exogenous auxin treatment. This is consistent with the long-standing notion that auxin drives dynamic developmental outcomes within primary roots (3, 8, 65–67), and we propose that early root morphogenesis events are shaped by distinct cellular proteomes.

Previous proteome studies based on auxin responses in seedlings and roots involved older seedlings and later time points compared with this study (10, 11). In Slade *et al, (*11), the authors examined auxin-mediated proteome changes in young seedlings at 8, 12, and 24 h after exogenous auxin treatment and thus captured auxin-regulated proteins associated with root differentiation. Because these are later time points than what we sampled here, it is difficult to directly compare the results between these studies. However, we did examine the overlap between these studies and found several proteins in common that are differentially regulated in the root following auxin treatment (Table S3). Altogether these proteins may represent a set of auxin biomarkers that are rapidly and stably expressed following auxin treatment and are reproducibly detected via peptide mass spectrometry. They include proteins such as SNX1 and nitrilase 1; collectively these proteins play important functional roles in various aspects of auxin transport, signaling, and biosynthesis (12, 42–46).

In addition to global characterization of auxin-regulated proteins, we developed targeted proteomics assays to simultaneously quantify all six endogenous auxin receptors. These targeted assays both validated and extended our iTRAQ studies. All six auxin receptors (TIR1 and AFB1–5) appear to have stable protein levels at both 30 min and 120 min following auxin treatment. Additionally, these targeted proteomics assays provide a novel method for quantification of auxin receptors from *in vivo* tissues, which could be applied to several biological questions related to auxin signaling, including natural variation and parameterization of existing mathematical models.

This study sought to describe how early auxin-signaling events influences cellular proteomes in organ-specific context. Hundreds of proteins change rapidly in response to auxin in roots, including cell wall modification enzymes. We examined the role of one such protein, GAUT10, in auxin-mediated root development using genetic analyses. In our proteomic profiling, GAUT10 is downregulated at both 30 min and 120 min following exogenous auxin treatment in roots. Loss of function alleles of *gaut10* have short roots that are exacerbated when grown without sucrose. Auxin response assays demonstrated that *gaut10* roots can respond normally to auxin when grown in the presence of sucrose. The short root phenotype is attributed to a reduction in root apical meristem size. Because GAUT10 is a glucuronosyltransferase, we hypothesize that *gaut10* mutants are hypersensitive to low sucrose conditions due to the function of this enzyme to modify pectin composition via attachment of sugar moieties. Notably, several other root mutants involved in auxin signaling also exhibit a similar sugar-dependent phenotype (63, 68–70). This includes *gin2* (Hexokinase) and MEDIATOR *med 12 med13* double mutants (71, 72). Additionally, sugar signaling has been shown to positively affect root growth via auxin (72), and transcriptional studies indicate that glucose can affect the expression of auxin biosynthesis genes, PIN transporter proteins, and several genes involved in auxin signaling (72). Additionally, glucose has been proposed to control nontranscriptional processes such as protein stability. Auxin and glucose act agonistically to activate TOR kinase activity (73, 74) while a large portion of auxin-regulated genes are antagonistically regulated by glucose (72), indicating that there is much to still understand related to these complex signaling pathways. Further genetic and molecular studies will be required in order to examine possible molecular links between GAUT10 activity, nutrient sensing, and auxin signaling.

Altogether, these datasets provide a rich resource for mining novel protein function. In particular, numerous proteins show significant altered abundance levels in a temporal fashion that makes these excellent candidates for future functional studies. Additionally, these datasets can inform new hypotheses of what biological processes may govern rapid auxin responses downstream of perception, including complex levels of gene regulation and rapid alteration of metabolic states.

## DATA AVAILABILITY

All supporting data have been deposited to the MassIVE repository developed by the National Institutes of Health-funded University of California San Diego Center for Computational Mass Spectrometry. The data obtained are available at MassIVE (MassIVE ID MSV000079857): https://massive.ucsd.edu/ProteoSAFe/static/massive.jsp.

## Supplementary Material

Table S5

Table S4

Table S3

Table S2

Table S1

Supplementary Figures

Table S6

## Footnotes

^1^ The abbreviation used is:

TIR1: transport inhibitor response 1.
