# Supplementary material for: Quantitative Early Auxin Root Proteomics Identifies GAUT10, a Galacturonosyltransferase, as a Novel Regulator of Root Meristem Maintenance
Source: Mol Cell Proteomics. 2019 Mar 27;18(6):1157–70. doi: 10.1074/mcp.RA119.001378 (PMC6553934; doi:10.1074/mcp.RA119.001378)
Supplement: Supplementary Figures [file 143649_0_supp_283079_pgcxgw.pdf]

Supplementary Material

Table S1. Auxin responsive proteomes of Arabidopsis roots at 30 min and 120 min (2h). iTRAQ intensities, log2 ratios and *P*-values for all proteins detected. Proteins with a *P*-value ≤ 0.05 were considered to be significant and are listed as separate sheets within the file. Data analysis was performed using Perseus as described in the methods.

Table S2. GO enrichment analyses performed on differentially expressed proteins using Panther identified several biological processes enriched at both 30 min and 120 min following auxin treatment in roots.

Table S3. Proteins in common between this study and another recently published auxin proteome study by (11).

Table S4. Primers used in this study for genotyping and RT-PCR.

Table S5. Peptide sequences used for targeted proteomics assays for auxin receptor proteins.

Table S6. Annotated spectra need for the 95 proteins with single-peptide identifications from Table S1.

Figure S1. Auxin regulated proteins are enriched in several gene ontology (GO) biological process categories as determined by STRING analysis. (A) Hierarchical clustering of GO functional categories enriched in differentially expressed (DE) proteins following 30 min and 120 min of auxin treatment in roots. Categories in common to both time points include amino acid metabolism and translation. 'Positive regulation of microtubule polymerization or depolymerization' is enriched only in the 30 min DE proteins while 'thigmatropism' is only enriched in the 120 min DE proteins.

Figure S2. Multiple reaction monitoring (MRM) analysis of TIR1/AFB proteins. Plants were either treated with a mock (M) solution or 1 μM IAA for 30, 60, or 120 min. The abundance of each protein was determined by first scaling the light (endogenous) peptide intensity relative to a corresponding heavy labeled spike in peptide and then normalizing to stably expressed ACTIN. Data are means of 3 or 4 independent biological replicates ± SEM. The values above the bars represent the *P* value for each comparison as determined by *t* tests.

Figure S3. Skyline chromatograms for quantified multiple reaction monitoring (MRM) analysis of TIR1/AFB proteins. Intensity is on the y-axis and retention time is on the x-axis. Reference peptide ("heavy") signals are in red traces while endogenous ("light") are in blue traces. The peptide sequence is shown for each target protein. (A) Actin peptide sequence is VAPEEHPVLLTEAPLNPK and it recognizes the following ACTIN proteins: AT2G42170, AT5G59370, AT3G46520, AT3G12110, AT5G09810. (B) LNVEVIDER from TIR1 (C) SSSLEEIR from AFB1 (D) DSPFGDTALLADVSK from AFB2 (E) DSPFGNAALLADVGR from AFB3 (F) ALEELVVR from AFB4 (G) LAVSGLLTDQAFR from AFB5. (H) Chimeric sequence of unique tryptic peptides designed for actin (grey), TIR1 (red), AFB1 (pink), AFB2

(green), AFB3 (orange), AFB4 (blue), and AFB5 (cerulean) shown with predicted cleavage sites as forward slashes; this sequence was synthesized and cloned into a pUC57 backbone.

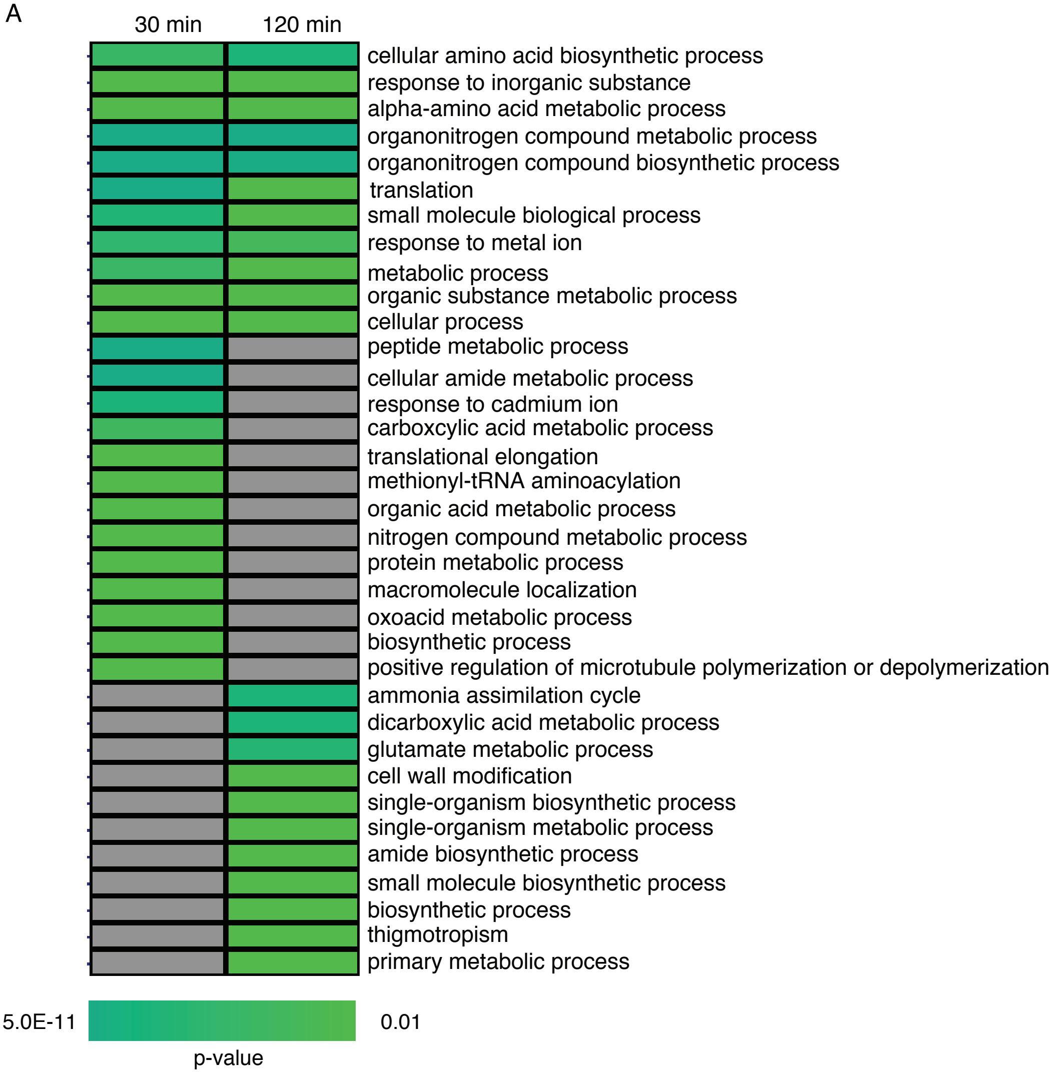

Figure S1. Auxin regulated proteins are enriched in several gene ontology (GO) biological process categories as determined by STRING analysis. (A) Hierarchical clustering of GO functional categories enriched in differentially expressed proteins (DE) following 30 min and 120 of auxin treatment in roots. Categories in common to both time points include amino acid metabolism and translation. 'Positive regulation of microtubule polymerization or depolymerization' is enriched only in the 30 min DE proteins while 'thigmatropism' is only enriched in the 120 min DE proteins.

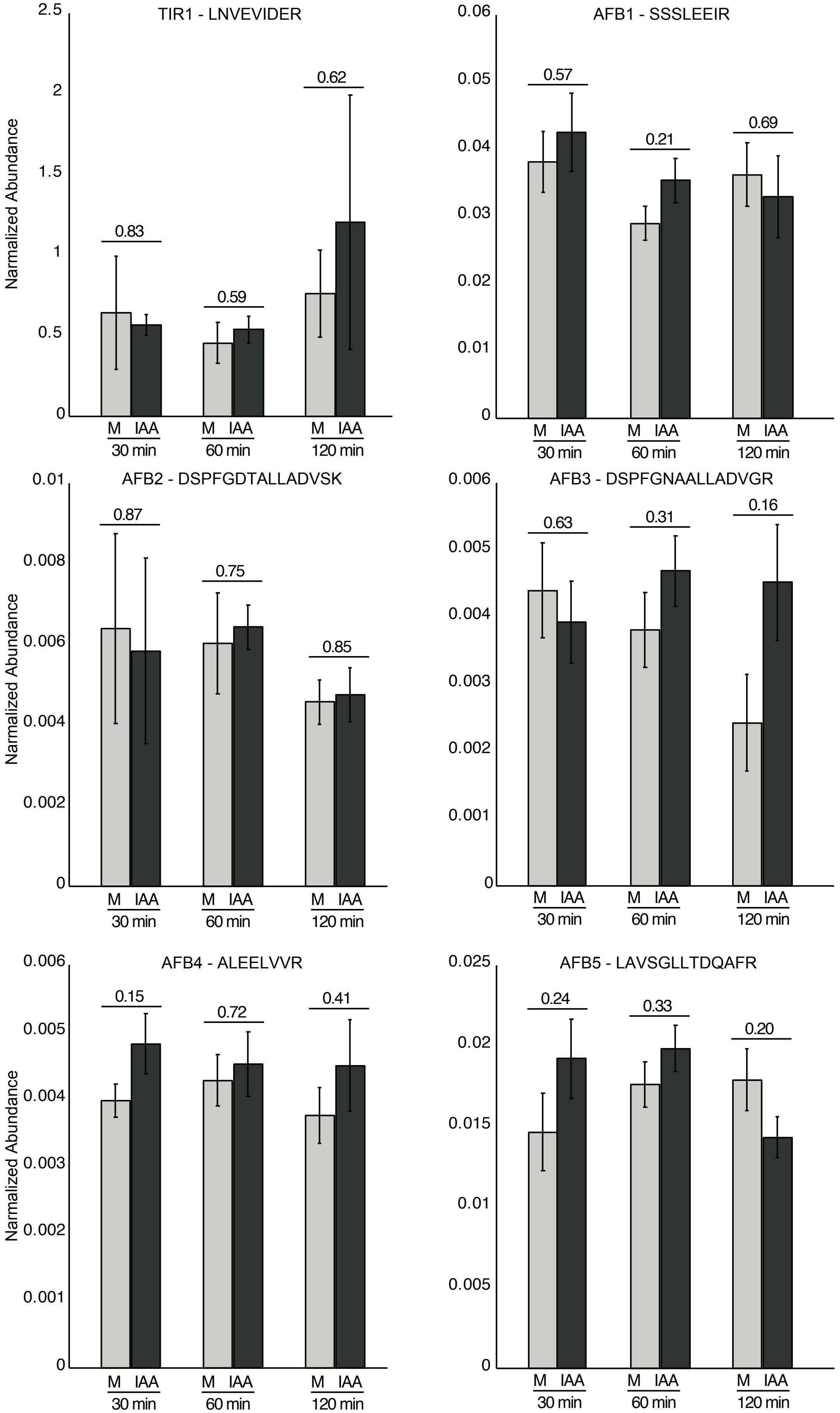

Figure S2. Multiple reaction monitoring (MRM) analysis of TIR1/AFB proteins. Plants were either treated with a mock (M) solution or 1 mico molar IAA for 30, 60, or 120 min. The abundance of each protein was determined by first scaling the light (endogenous) peptide intensity relative to a corresponding heavy labeled spike in peptide and then normalizing to stably expressed ACTIN. Data are means of 3 or 4 independent biological replicates ± SEM. The values above the bars represent the P value for each comparison as determined by t tests.

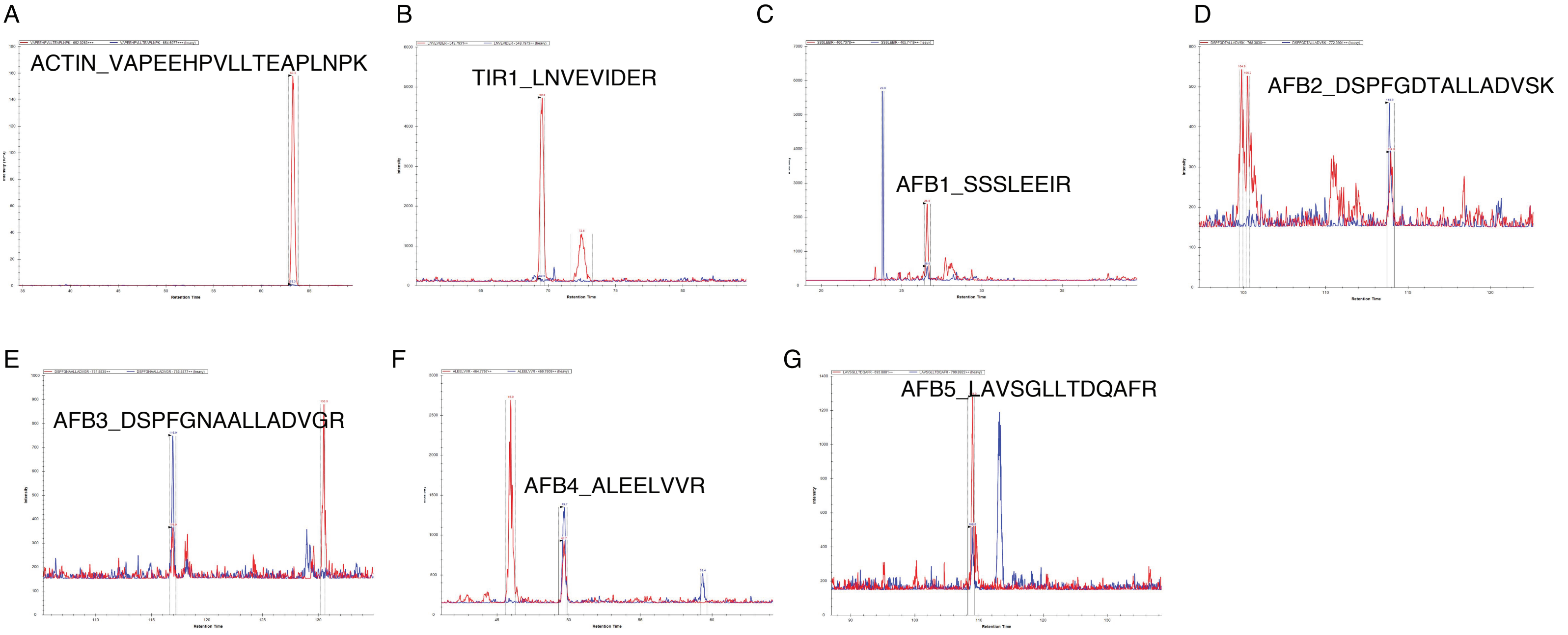

Figure S3. Skyline chromatograms for quantified multiple reaction monitoring (MRM) analysis of TIR1/AFB proteins. Intensity is on the y-axis and retention time is on the x-axis. Reference peptide ("heavy") signals are in red traces while endogenous ("light") are in blue traces. The peptide sequence is shown for each target protein. (A) Actin peptide sequence is VAPEEHPVLLTEAPLNPK and it recognizes the following ACTIN proteins: AT2G42170, AT5G59370, AT3G46520, AT3G12110, AT5G09810. (B) LNVEVIDER from TIR1 (C) SSSLEEIR from AFB1 (D) DSPFGDTALLADVSK from AFB2 (E) DSPFGNAALLADVGR from AFB3 (F) ALEELVVR from AFB4 (G) LAVSGLLTDQAFR from AFB5.
